# Navigation of a magnetic micro-robot through a cerebral aneurysm phantom with magnetic particle imaging

**DOI:** 10.1038/s41598-021-93323-4

**Published:** 2021-07-07

**Authors:** Anna C. Bakenecker, Anselm von Gladiss, Hannes Schwenke, André Behrends, Thomas Friedrich, Kerstin Lüdtke-Buzug, Alexander Neumann, Joerg Barkhausen, Franz Wegner, Thorsten M. Buzug

**Affiliations:** 1 Institute of Medical Engineering, University of Lübeck, Ratzeburger Allee 160, 23562 Lübeck, Germany; 2 Fraunhofer Research Institution for Individualized and Cell-Based Medical Engineering IMTE, Mönkhofer Weg 239a, 23562 Lübeck, Germany; 3 Department of Neuroradiology, University Hospital Schleswig-Holstein, Campus Lübeck, Ratzeburger Allee 160, 23562 Lübeck, Germany; 4 Department of Radiology and Nuclear Medicine, University of Lübeck, Ratzeburger Allee 160, 23562 Lübeck, Germany

**Keywords:** Biomedical engineering, Experimental models of disease, Three-dimensional imaging, Magnetic devices

## Abstract

Cerebral aneurysms are potentially life threatening and nowadays treated by a catheter-guided coiling or by a neurosurgical clipping intervention. Here, we propose a helically shaped magnetic micro-robot, which can be steered by magnetic fields in an untethered manner and could be applied for a novel coiling procedure. This is shown by navigating the micro-robot through an additively manufactured phantom of a human cerebral aneurysm. The magnetic fields are applied with a magnetic particle imaging (MPI) scanner, which allows for the navigation and tomographic visualization by the same machine. With MPI the actuation process can be visualized with a localization accuracy of 0.68 mm and an angiogram can be acquired both without any radiation exposure. First in-vitro phantom experiments are presented, showing an idea of a robot conducted treatment of cerebral aneurysms.

## Introduction

Magnetic actuation (MA) is of great potential for a variety of different application scenarios. The advantage of applying external magnetic fields to steer a robot is that no onboard energy supply is needed and miniaturization towards the micrometer scale becomes possible. Steerable capsules for the inspection of the gastrointestinal tract^1,2^, millimeter-sized robots for vascular applications^3–5^ and micrometer-sized helical swimmers^6,7^ have been already developed. They enable targeted drug delivery^8,9^ and can be deployed inside very small cavities, such as the eye^10,11^. However, the tomographic visualization in real-time remains challenging. Medical imaging techniques such as X-ray-based modalities, Magnetic Resonance Imaging (MRI), Ultrasound or PET have been investigated, but they show disadvantages in either spatial or temporal resolution or utilize ionizing radiation^9,12,13^.

Magnetic Particle Imaging (MPI) appears as a highly promising imaging modality for tracking the movement of magnetic robots^13^. MPI is a medical imaging technique, which has been intensively developed within the last fifteen years and is nowadays at the stage of preclinical investigations. It utilizes the nonlinear magnetization behavior of superparamagnetic iron oxide nanoparticles (SPIONs) and visualizes their concentration distribution^14^. MPI is of great interest for vascular interventions^15^ and neuroimaging^16^. A beating mouse-heart as well as SPION-coated interventional devices have been visualized so far^17,18^. The advantage of combining MA with MPI is that the magnetic fields generated by an MPI scanner allow MA as well, therefore no imaging modality has to be integrated into an actuation setup or vice versa, despite that MPI is capable of tomographic real-time imaging without the need of ionizing radiation and nephrotoxic contrast agents^17^. An MPI scanner induces a magnetic force by its gradient field, such that magnetic devices^19,20^ or particles^21,22^ can be moved. Furthermore, a rotation of magnetic devices or particles can be generated by using the homogeneous magnetic fields of the focus fields and applying alternating currents to the coils resulting into a rotating field vector^4,23,24^. In comparison, MRI machines also feature a magnetic gradient field, which can be utilized for MA. However, the gradient strength is smaller and therefore, the force applied is weaker. Furthermore, rotating magnetic fields cannot be applied with an MRI machine and SPIONs are visualized as a cancellation of signals in MRI. Hence, the localization can be imprecise, and a quantification of tracer material is not possible.

The navigation of robots towards a targeted position in an untethered manner opens the possibility to reach regions of the body, which are difficult to access even by interventional techniques. Nowadays, endovascular interventions are increasingly replacing surgical procedures as they are related to less (peri)-operative complication rates and faster rehabilitation of the patients. Thus, catheter-guided endovascular interventions have become the standard therapy for the treatment of numerous vascular diseases. Major parts of the vascular system can be reached minimal invasively including a wide range of therapeutic options, e.g. balloon dilatation and stent implantation, thrombectomy of occluded vessels and embolization/coiling of aneurysms.

Typical localizations of aneurysms are the cerebral arteries. Between 3 and 5% of the population carry cerebral aneurysms^25^ with a rupture rate of 0.95%^26^. The rupture of a cerebral aneurysm is potentially life threatening. Cerebral aneurysms are often incidental findings or are diagnosed as the origin of severe intracranial bleeding. Next to minimal surgical clipping, they are treated by inserting metallic coils or intrasaccular embolization devices during a catheter-guided intervention. The most critical part of these interventions is the advancement of the microcatheter into the aneurysm, which can cause life-threatening complications, especially in the case of aneurysms located far peripherally and individual anatomies that are difficult to probe. Skill and experience of the interventionalist are crucial for the success of the procedure and many complications, such as perforation of the aneurysm wall or inadvertent occlusion of the carrier vessel are often due to human error. In a highly technological future, it may be possible to eliminate such sources of error and replace the critical process of catheter-based aneurysm probing and catheter-based aneurysm embolization with the use of remote-controlled micro-robots.

Here, we introduce a magnetic micro-robot, which can be steered through a phantom of a human cerebral aneurysm and can be localized with high accuracy by using an MPI scanner, which demonstrates the potential application of conducting aneurysm coiling in an untethered manner. The micro-robot is additively manufactured and coated with magnetic particles. The latter allows both MA with rotating focus fields of a preclinical MPI scanner and visualization with MPI. This is a new approach for the interventional treatment of cerebral aneurysms without the use of iodine-based contrast agent and ionizing radiation.

## Results

### The magnetic micro-robot

**Figure 1:**
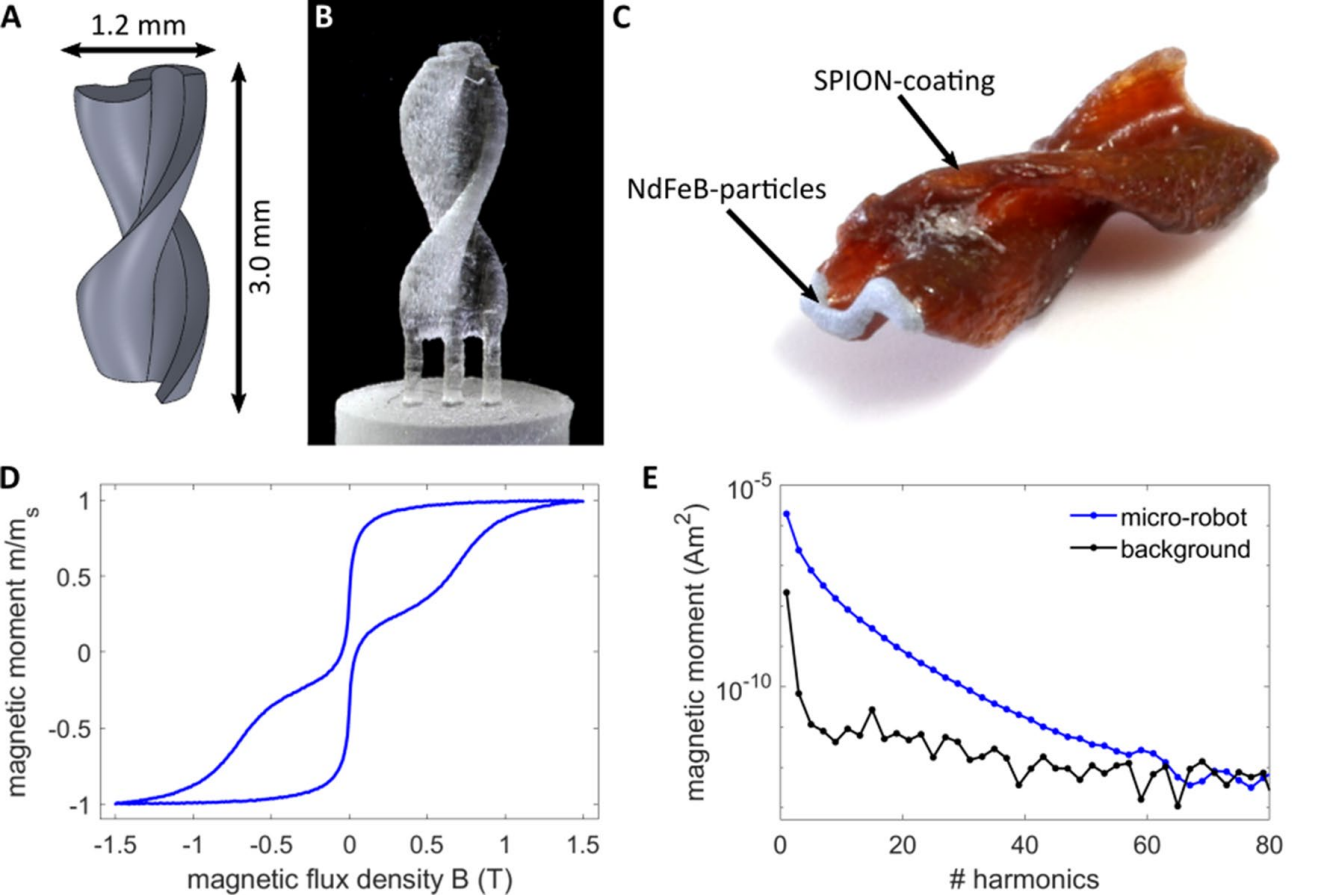
The magnetic micro-robot. (**A**) The CAD model of the micro-robot. The geometry is inspired by vertical axis wind turbines and is called helical Savonius shape. (**B**) The micro-robot is 3D-printed using a stereolithographic printing procedure. (**C**) It is coated with SPIONs for the visualization with MPI. The tip consists of NdFeB particles, introducing a magnetic moment for MA. (**D**) The magnetization curve of the micro-robot, acquired with a VSM showing a superposition of a paramagnetic and a ferromagnetic behavior. (**E**) The odd harmonics of the amplitude spectrum, acquired with a magnetic particle spectrometer with an excitation frequency of 25 kHz and an amplitude of 20 mT.

The magnetic micro-robot (see Fig. 1A–C) has a helical Savonius shape and is coated with SPIONs for the visualization with MPI. The shape is adopted from vertical axis wind turbines^27^ and was already found to be suitable as a magnetic robot at larger scales^4^. The robot’s tip is painted with neodymium-iron-boron (NdFeB) particles to introduce a defined magnetic moment. Details about the manufacturing and coating of the robot can be found in “Materials and methods”. The magnetization behavior (Fig. 1D) shows two regimes of magnetization, a superparamagnetic and a ferromagnetic, corresponding to the two types of particles, which were measured with a vibrating sample magnetometer (VSM). The SPIONs are being magnetized at much lower field strengths than the NdFeB particles. The latter introduce a remanence. The amplitude spectrum measured with a magnetic particle spectrometer shows higher harmonics beyond the 50th harmonic, indicating the suitability of the micro-robot for MPI (Fig. 1E). Numerical simulations have been carried out on the fluid dynamics of the magnetic micro-robot (Fig. S1). They show a velocity of about (1.56 ± 0.11) mm/s at a rotation frequency of 5 Hz inside water. Experimental results show an increasing velocity as a function of the applied rotation frequency until a certain step-out frequency, where the synchronous regime of motion passes over into a slip-stick motion. The point of transition depends on the applied field amplitude (Fig. S2). Details can be found in the Supplementary Material.

### The aneurysm phantom

**Figure 2:**
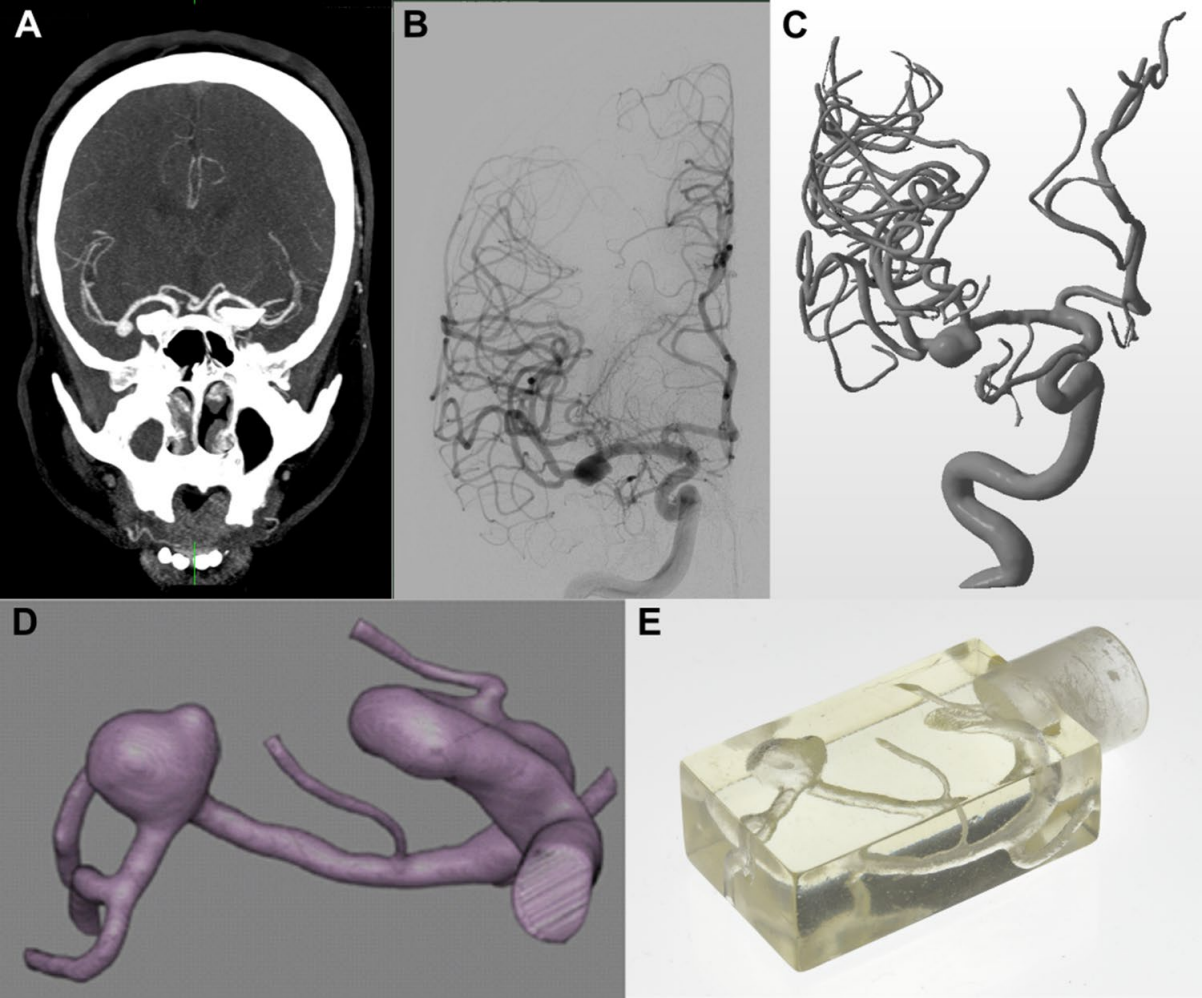
Aneurysm phantom. The aneurysm is located at the right middle cerebral artery and indicated by the blue arrow. (**A**) The patient’s CT image. (**B**) One image from patient’s 3D rotational angiography images. (**C**) 3D rendered representation of the patient’s rotational angiography. (**D**) For further processing, the aneurysm and the access route were isolated as shown by the coronary oblique view. (**E**) The 3D-printed phantom is enclosed by a cuboid and can be attached to the sample holder of the MPI scanner by a plug-in connection.

A patient-representative 3D-printed model of the right middle cerebral artery was produced, which shows an aneurysm with maximum diameters of 7.24 mm, 5.37 mm and 6.12 mm in each spatial direction, respectively. The data was acquired by using a 3D rotational angiography scan with a slice thickness of 0.238 mm (see Fig. 2). More detailed information on the data acquisition can be found in “Materials and methods”.

### Tracking accuracy for the micro-robot

**Figure 3:**
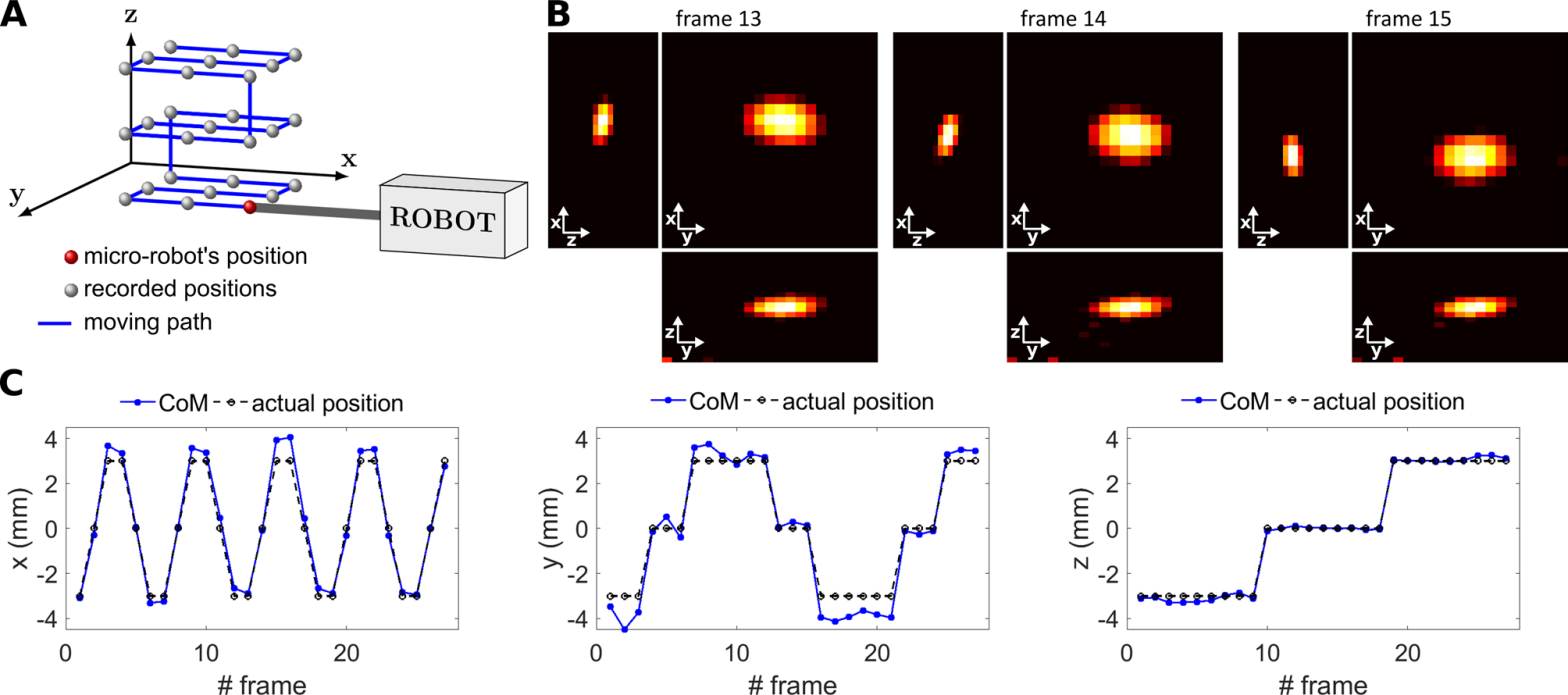
Tracking accuracy for the micro-robot with MPI. (**A**) Robot movement for the acquisition of 27 spatial positions of the micro-robot. (**B**) Visualization of the micro-robot exemplarily at three different positions. Projection images for each spatial direction are shown. (**C**) The center-of-mass (CoM) calculation for 27 different positions, compared to the actual position of the micro-robot. A mean deviation of 0.68 mm and maximum deviation of 1.5 mm between the CoMs and the actual position were found.

To measure the accuracy for a later tracking of the micro-robot, it was attached to a sample holder and images of 27 spatial positions were acquired. Details can be found in “Materials and methods”. The visualization of the micro-robot as well as the center-of-mass calculations of the images can be seen in Fig. 3. From these measurements it can be stated that the micro-robot can be tracked with an average accuracy of 0.68 mm with a standard deviation of 0.35 mm, which corresponds to less than the voxel size ($2 \times 2 \times 1$ mm^3^) in the experiments carried out. A maximum deviation between the center-of-mass calculation and the actual position of the micro-robot was found to be 1.5 mm.

### Navigation of the micro-robot

**Figure 4:**
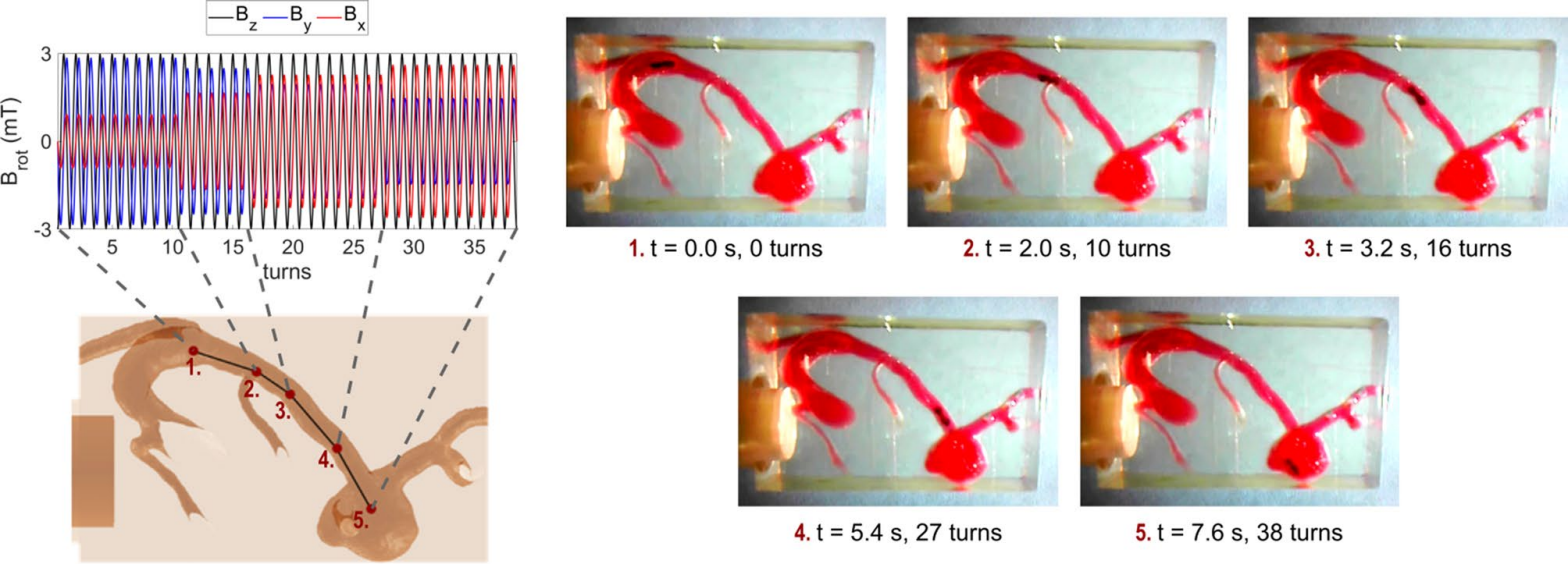
Navigation of the micro-robot with an MPI scanner through the aneurysm phantom. The path through the aneurysm phantom is approximated by four straight path segments by using the CAD model. According to the path segments the focus fields are calculated, such that the field vector rotates in the plane perpendicular to the path segment. A rotation frequency of 5 Hz and an amplitude of 3 mT is applied. The video stills show the resulting movement of the micro-robot through the human phantom, which was filled with a red stained glycerol-water mixture to mimic the viscosity of blood. The measurement is conducted inside an MPI scanner. The video can be found in the Supplementary Material.

A magnetic object carrying a magnetic moment $\overrightarrow{m}$ experiences a torque of the form $\overrightarrow{\tau}=\overrightarrow{B}\times \overrightarrow{m}$ inside a magnetic field $\overrightarrow{B}$, i.e. the magnetic moment aligns with the magnetic field vector. For navigating the micro-robot on an intended pathway, a homogeneous magnetic field with a rotating field vector is needed. The micro-robot follows the rotation and performs a forward velocity due to its shape. The field vector is rotated in the plane perpendicular to the intended direction of movement. Such a magnetic field can be generated with an MPI scanner by using the focus fields, which are typically used to shift the field of view (FOV) in space. The focus field consists of three coils, each generating a homogeneous magnetic field with a magnetic field vector orthogonally to each other. By the superposition of the three focus fields in x-, y- and z-direction, an arbitrary resulting field vector can be generated. When applying sinusoidal currents with a phase shift of 90° to the focus field coils, a rotating field vector can be generated. Detailed information can be found in “Materials and methods” and a schematic drawing is shown in Fig. 8. The pathway through the aneurysm phantom is discretized into four straight path segments, which seemed to approximate the actual path curvature sufficiently, and converted into corresponding magnetic field strengths for the focus field coils (see Fig. 4). The number of turns for each segment was calculated based on the experimentally observed velocity, which was found to be suitable to cover the path length. Since the used MPI scanner allows to read in discretized values of the focus fields, the calculated field sequences are discretized into 20 steps per rotation. The video, showing the micro-robot moving through the middle cerebral artery into the aneurysm of the phantom can be found in the Supplementary Material, selected frames of the video are displayed in Fig. 4.

### Tomographic visualization and navigation

As in clinical procedure, an angiogram of the aneurysm phantom is conducted at first. Here, the angiogram is recorded using MPI and a homogeneous tracer distribution filled into the phantom. Parameters for image acquisition, reconstruction and visualization are given in “Materials and methods”.

**Figure 5:**
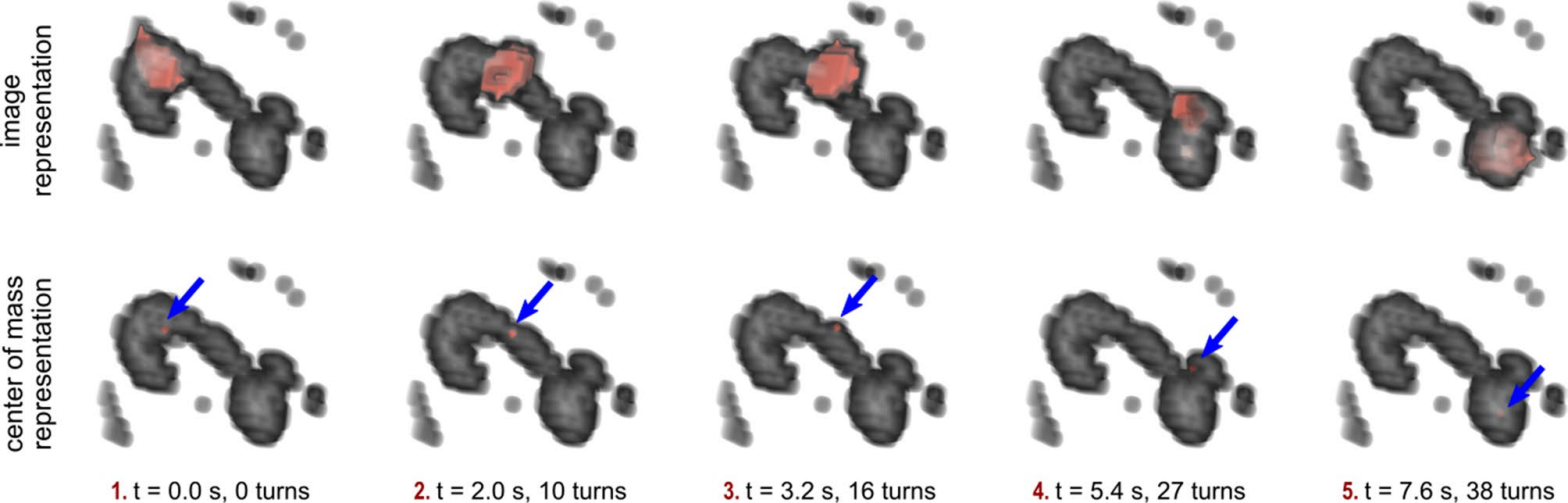
MPI images of the micro-robot inside the aneurysm phantom. Superposition of the reconstructed and registered images of the angiogram and the micro-robot at five positions along the pathway through the middle cerebral artery (see Fig. 4 for path segments). In the bottom row, the calculated center of mass of the micro-robot is visualized.

For tracking the micro-robot during MA, sequential images were acquired at the beginning and after each manipulation step resulting into five recordings (see parameters in “Materials and methods”). It has to be noted, that the aneurysm phantom was not filled with tracer material for MA but with the same glycerol–water mixture as for the navigation experiments. The reconstruction results of the micro-robot inside the aneurysm phantom can be seen in Fig. 5. An animated view can be found in the Supplementary Material. The reconstructed micro-robot is superposed with the reconstructed image of the angiogram (first row). Furthermore, the calculated center of mass of the micro-robot is superposed with the angiogram (second row) for demonstrating the tracking ability of the micro-robot.

### Hyperthermia of micro-robot

**Figure 6:**
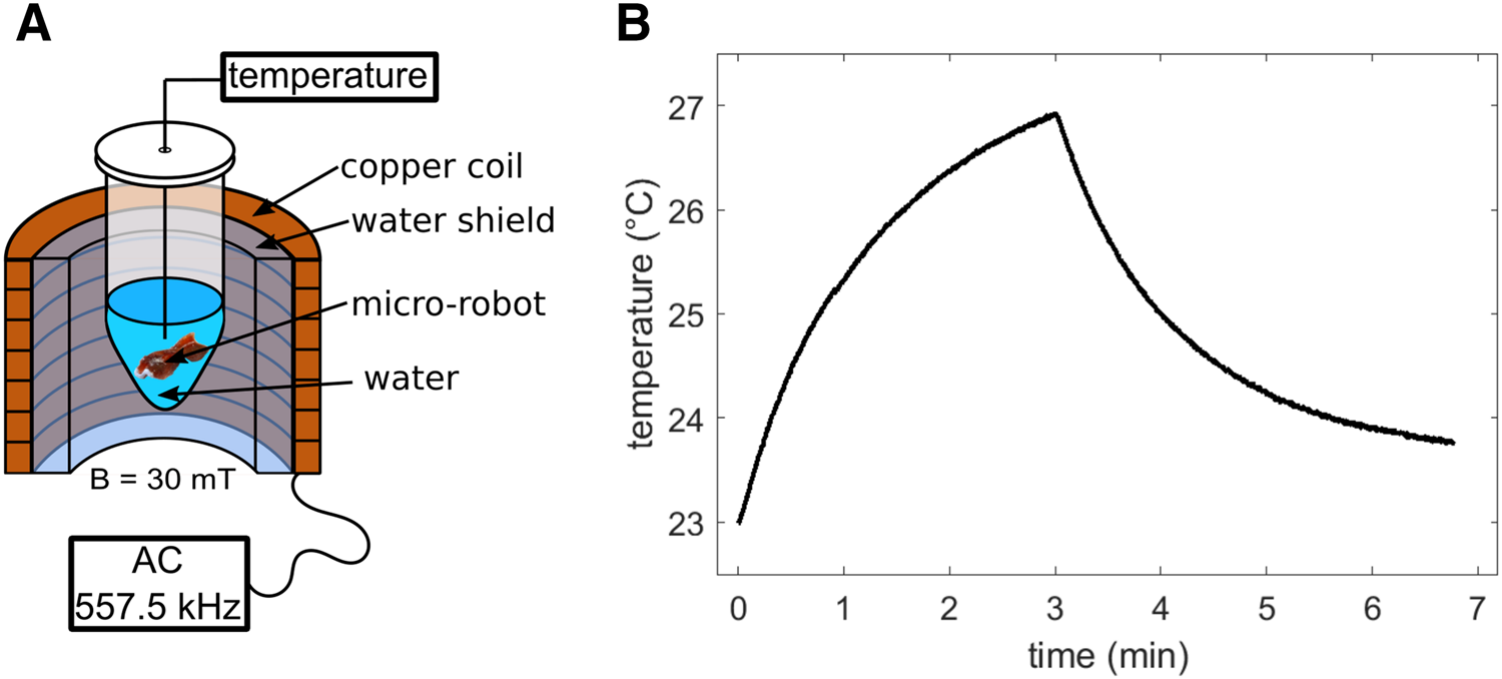
Hyperthermia measurement. (**A**) A schematic drawing of the hyperthermia setup. The micro-robot was covered with water inside an Eppendorf tube. An oscillating magnetic field of 30 mT amplitude and 557.5 kHz frequency was applied for 3 min. (**B**) The temperature of the water, surrounding the micro-robot is displayed.

A fluid, which contains magnetic nanoparticles, can be heated by applying fast alternating magnetic fields. This technique is widely used for therapeutic reason to locally heat abnormal tissue^28^. Since heat facilitates the clotting of blood it is aimed at increasing the temperature inside the aneurysm. A temperature increase of about 4 K was observed in the sample chamber within a heating time of 3 min, before the sample cooled down when switching off the field. The measurement results can be seen in Fig. 6.

## Discussion

In this work we show the potential of MPI for the steering and visualization of a micro-robot with the perspective of cerebral aneurysm coiling. Our previous work already showed the feasibility of a 2D visualization and actuation of a coated swimmer with MPI^4^. Here, we improved the manufacturing process of the micro-robot in terms of smaller size (because another 3D-printing technique was used) sufficient imaging signal even though a smaller object provides less surface for coating and larger magnetic moment (because another coating procedure and different particles were used). The larger magnetic moment can be assumed as a much lower field strength led to a rotation of the micro-robot (here 3 mT, in^4^ 18 mT). Further, the new opportunity of path planning allowed the steering through a model of a real vascular structure. The investigation of the tracking accuracy, the 3D visualization with the overlay of an angiogram as well as the ability to conduct hyperthermia show the potential for future medical applications. In the last decade, numerous studies have proven the potential of MPI guided cardiovascular interventions. For example, a balloon-angioplasty was monitored in real time with MPI^29^. Furthermore, safety measurements of endovascular stents^30^ and catheters/guidewires^31^ were conducted. Flow measurements of aneurysms were given^32^ in a comparison study with digital subtraction angiography (DSA) and magnetic resonance angiography (MRA). Furthermore, the detection of ischemic stroke and cerebral bleeding in rodents is possible with MPI^33,34^. Thus, in principle MPI seems to be eligible to conduct monitoring of aneurysm coiling. Especially due to the absence of nephrotoxic contrast agents and ionizing radiation for the acquisition of the angiogram, MPI is a very promising alternative to the clinically established DSA. Clinical MPI scanners are not available yet, but a first human sized MPI scanner for brain application was presented^35^. This scanner already features a FOV which would be large enough to image the cerebral vessels of a single hemisphere. So far, perfusion imaging was sufficiently performed with this scanner utilizing in-vitro flow models.

The used phantom is a representation of a patient’s anatomy. However, the used material and the conditions are not directly comparable. The phantom was filled with a glycerin-water mixture and the experiments were conducted without flow. In a subsequent in-vivo procedure it could be possible to stop the blood flow by means of a balloon catheter to achieve significantly improved control of the microrobot, which should both improve the accuracy of the procedure and increase patient safety by reducing embolic complications. Further, it needs to be mentioned that the navigation of the here presented micro-robot is limited in terms of uphill and downhill navigation. It can overcome some obstacles and smooth ascents, but for navigating against the gravity a micro-robot of larger buoyancy would be needed or much larger rotation frequencies need to be applied.

The micro-robot may get washed away by the blood flow causing embolic complications as soon as it has reached the aneurysm and the rotating magnetic fields are turned off. To hold the micro-robot in its designated position until a sufficient thrombosis of the aneurysm occurs it is possible to switch on the MPI scanner’s gradient field. It induces a magnetic gradient field, which applies a force and can therefore hold the micro-robot inside the aneurysm, where it remains. One might also think of the development of a micro-robot carrying a coil, which is made off a micro platinum wire and can be detached as soon as a proper placement is achieved.

The acquisition of an angiogram and the tracking of the micro-robot are feasible with a satisfying accuracy of less than 1 mm, which is reasonable for the intended application as the middle cerebral artery diameter in humans is ~ 2.5 mm^36^. Furthermore, the achieved imaging accuracy is in the range of the spatial resolution of MRA, but less than DSA^37^. If necessary, the tracking accuracy could be further improved by using a gradiometric receive coil^38^. For the investigation of the accuracy and reliability of the steering process a systematic study inside dedicated phantoms and under in-vivo conditions need to be conducted in future. In-vivo, a bolus of SPIONs would be administered for the acquisition of an angiogram. For a real-time tracking the sequences between imaging and actuation need to be altered much faster, which is a constraint of the used MPI scanner. Nevertheless, the perspective of this approach should be a closed loop control of the micro-robot.

The therapeutic effect of aneurysm coiling is achieved by cutting off the blood supply from the carrier vessel into the aneurysmal lumen by filling the aneurysm with embolization coils, whereby more than one coil is usually required for complete aneurysm occlusion. In our study we used a single micro-robot to illustrate the potential for aneurysm coiling, where the aneurysm phantom would have required multiple conventional coils using standard endovascular treatment. To achieve complete aneurysm occlusion in this case, several micro-robots would have to be used and ideally functional coating material should be applied. The use of a coating^39^ or 3D-printing material^40^ that swells in liquid surrounding could be an option or a thrombogenic coating of the micro-robot could be an approach to improve the embolization effect. The feasibility of the micro-robot to conduct hyperthermia opens the opportunity to apply heat to the surrounding of the micro-robot as soon as it has reached the aneurysm to induce blood coagulation faster^41^. Local temperatures of 42 °C are necessary^9^, which are potentially applicable with the micro-robot since a temperature difference of 4 °C was already achieved after 3 min of heating. In relation to a core body temperature of 38 °C the measured temperature increase thus should be sufficient to induce blood coagulation. Furthermore, the heating of the micro-robot could be used as a trigger for the release of a thrombogenic drug coating to ensure the embolizing effect of the swimmer^42,43^. However, heat perfusion needs to be considered for in-vivo treatments and the nanoparticles used for the coating could be optimized for the induction of heat. It has been shown previously that it is possible to monitor the heat transfer with MPI^44–46^. Recently, a hyperthermia insert has been presented, which can be attached to the used MPI scanner and allows the alternation of heating and visualization in one machine^47^. Hence, the here presented study is pointing the way towards MA, hyperthermia and visualization with MPI.

There are some limitations of our work which should be addressed: the overall biocompatibility of the micro-robot has not been investigated and was not in the focus within the presented study, the used material NdFeB and the impregnating agent of the coating are non-biocompatible ingredients. Further, studies need to be conducted which show the effectiveness of the micro-robot compared to nowadays used coiling procedures. Especially, the use of multiple micro-robots should be investigated in future. Furthermore, in vivo studies are needed to prove the introduced concept under more realistic conditions. Especially the effect of the micro-robot on the wall of the vessel, where it is moving through, as well as on the aneurysm should be investigated in detail. In addition, the influence of the blood flow and the feasibility of balloon induced hemostasis should be investigated. In this study we focused on the investigation of the interaction of the micro-robot with a rotating magnetic field, its steerability through an anatomic vessel structure and the accuracy of localizing the micro-robot’s position with MPI.

## Conclusion

A magnetic micro-robot was developed, which can be navigated by rotating focus fields of an MPI scanner through the anatomy of a human middle cerebral artery towards an aneurysm. The aim of using the micro-robot as an untethered embolization device was presented using a human aneurysm phantom, which was additively manufactured from patient’s anatomy. A magnetic coating of the 3D printed micro-robot was developed, to induce magnetic properties for actuation and visualization with MPI. The micro-robot’s location can be tracked accurately with a mean deviation of 0.7 mm using a center of mass calculation. For steering the micro-robot into the aneurysm, the intended pathway was discretized into four path segments and the corresponding magnetic fields were programmed and applied with a preclinical MPI scanner. The micro-robot was successfully maneuvered on the intended pathway into the aneurysm. An angiogram of the phantom, which was filled with tracer material, was acquired with MPI, showing the middle cerebral artery and the aneurysm. Sequential images were acquired of the micro-robot, which have been superposed with the angiogram showing the stepwise movement using MPI. The presented phantom study opens a new idea for cerebral aneurysm coiling with a magnetic micro-robot, conducted and monitored by an MPI scanner.

## Materials and methods

### Manufacturing and coating of the magnetic micro-robot

**Figure 7:**
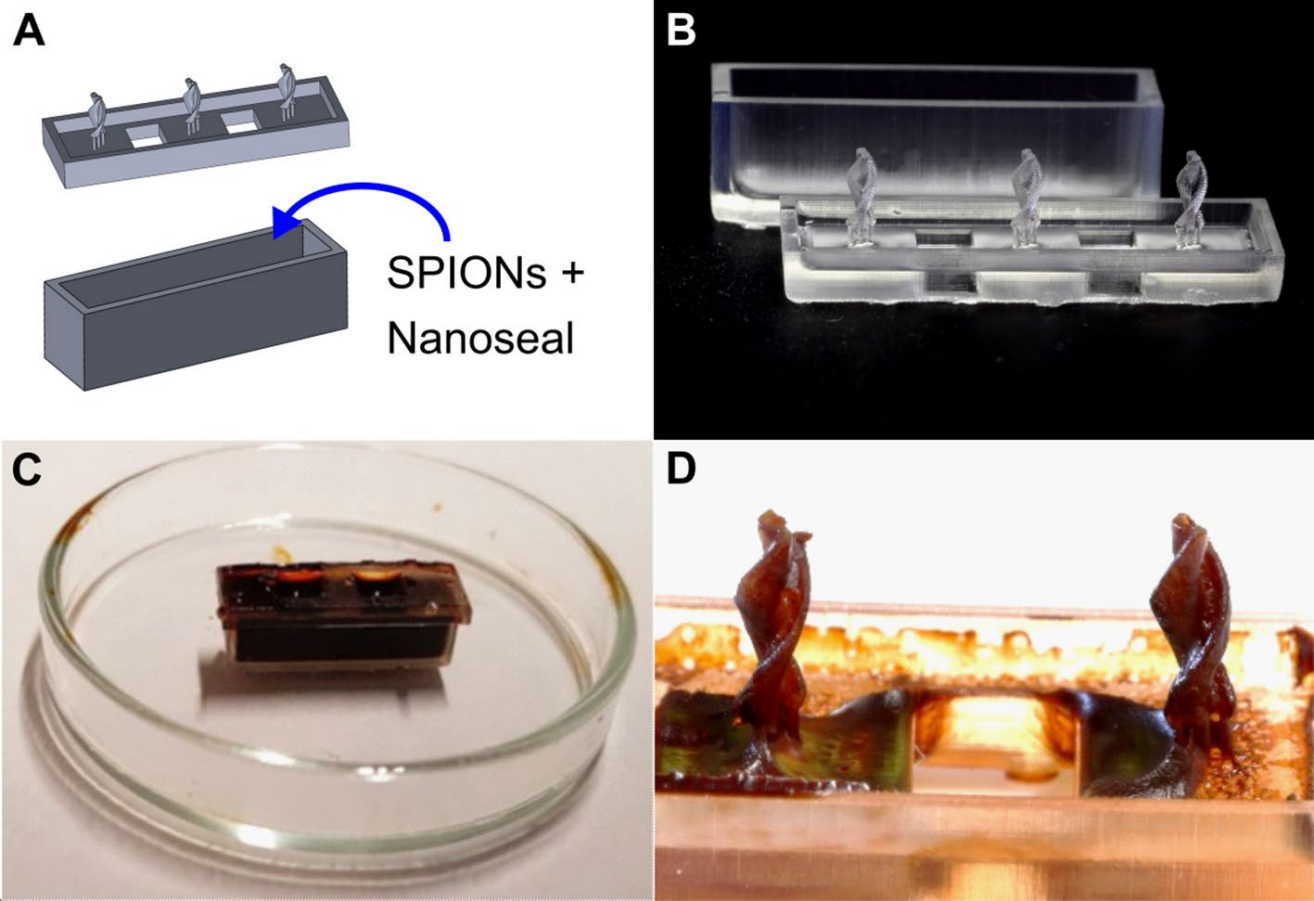
Coating procedure of the micro-robot. (**A**) CAD model of three micro-robots attached to a cap of a little basin. (**B**) 3D-printing of the micro-robots and the basin. (**C**) The tank was then filled with the SPION-impregnating agent suspension. The tank was closed with the cap, such that the micro-robots are dipped into the suspension. (**D**) The evaporation of the water in the suspension formed the SPION coating of the micro-robot. Afterwards the swimmers are being detached from the cap and the tip is painted with a NdFeB containing lacquer, as can be seen in Fig. 1.

The SPIONs for the coating of the micro-robots were produced by the coprecipitation method^48^. The solution had an iron content of 0.3 mol/l. 1 ml of the particle solution was mixed with 0.5 ml of an impregnating agent (Nanoseal 180 W, JELN Imprägnierung GmbH, Schwalmtal, Germany). A little basin with an inner volume of $(26\times 6\times 7)$ mm^3^ = 1.1 ml, was filled with the SPION-impregnating agent suspension. The magnetic micro-robot and the basin were 3D-printed (Form2, Formlabs Inc., Somerville, USA) with “High Temp Resin” (Formlabs Inc.). Three micro-robots were printed with three pillars each on top of the basin’s cap. When closing the basin, the micro-robots are dipped into the SPION-Nanoseal suspension. It was then put into the oven for 100 min at 60 °C. The water of the solution could evaporate through holes in the cap. Figure 7 illustrates the SPION-coating procedure. Further, NdFeB particles (5 µm Magnequench, Singapore) were mixed into an acrylic lacquer (Cryl Studio, Lukas, Nürnberg, Germany) with 20 wt%. The tip of the micro-robot was then painted with the NdFeB-lacquer. The micro-robot was immediately positioned onto the surface of a permanent NdFeB magnet during drying to align the magnetic moments of the particles.

### Characterizing the micro-robot’s magnetic behavior

The magnetic behavior was analyzed with a vibrating sample magnetometer (8607 VSM System, Lake Shore Cryotronics, Inc., Westerville, Ohio, USA). A magnetic field of up to 1.5 T was applied. The dynamic behavior was measured with a magnetic particle spectrometer, featuring a one-dimensional excitation of 25 kHz and a field amplitude of 20 mT with a spectral resolution of 2.5 kHz^49^. The odd-harmonics of the amplitude spectrum are displayed in Fig. 1E, which is the Fourier transform of the time dependent signal induced by the SPIONs’ nonlinear change of magnetization.

### The aneurysm phantom

The 3D clinical rotational angiography data was acquired using an angiography system (Allura Xper FD 20/20, Philips Healthcare, Best, Netherlands) with the following acquisition parameters: 5 s acquisition time, 220° rotation, 150 individual images at a frame rate of 30/s, $(15\times 48)$ cm^2^ detector FOV with a $512\times 512$ pixel acquisition matrix. The images were reconstructed with a soft tissue kernel of isotropic voxel size (edge length 0.238 mm).

Segmentation was performed with Analyze Pro 1.0 (AnalyzeDirect, Overland Park, Kansas, USA), while a semi-automated approach using a region growth algorithm was used. For this purpose, seed areas were defined at different points in the vessels. Small perforator arteries were removed if the diameter was smaller than 0.1 mm. The segmented anatomy was converted into stereolithography (.stl) format. A rectangular box with a size of $(36.5\times 13.1\times 22.2)$ mm^3^ was designed to enclose the target vessels of the anatomy and an adapter for the sample holder of the MPI scanner was attached to the model. Printing was performed on a stereolithography printer (Form2, Formlabs Inc.) using a 25 µm layer thickness and clear photopolymer resin (Formlabs Inc.). After the printing process and post-processing steps, the application of clear coat was performed^50^.

### Tracking accuracy for the micro-robot

With a preclinical MPI scanner (MPI 25/20FF, Bruker BioSpin, Ettlingen, Germany) a system matrix was acquired at first that enables posterior image reconstruction. Drive field amplitudes of 12 mT have been applied in x-, y-, and z-direction and a gradient strength of 0.625 T/m in x- and y-direction and 1.25 T/m in z-direction was set, leading to a drive field FOV of $(38.4\times 38.4\times 19.2)$ mm^3^. The FOV has been discretized in steps of 2 mm in x- and y-direction and 1 mm in z-direction, according to the size of the used point sample, resulting in a system matrix of $21\times 21\times 22$ voxels and $(42\times 42\times 22$) mm^3^, respectively. This way, an overscan of the system matrix was performed, such that the resulting FOV is larger than the drive field FOV to avoid potential artifacts from particles outside the drive field FOV^51^. A measurement sample of 4 µl (liquid volume) of SPION-Nanoseal coating was dried to get the same properties as for the micro-robot’s coating. The receive signal has been averaged 50 times.

The micro-robot (see “Manufacturing and coating of the magnetic micro-robot”) has been attached to a sample holder, which can be driven precisely by a robot within the scanner bore. MPI measurements of the micro-robot have been taken at 27 different spatial positions using a $3\times 3\times 3$ pattern with a spacing of 3 mm. The same drive field amplitudes and gradient strength as for the system matrix measurement were applied. The receive signal has been averaged 500 times.

Image reconstruction for the 27 spatial positions was performed using frequency components of the system matrix ≥ 80 kHz that feature SNR values above 3. An unregularized Kaczmarz algorithm with one iteration has been used. An intensity value threshold of 40% of the maximum value has been applied prior to visualization and center of mass calculation. Three of the reconstructed images are exemplarily displayed in Fig. 3A. The center of mass (CoM) of all reconstructed images was calculated using

$$\vec{r}_{{{\text{CoM}}}} = \frac{{\mathop \sum \nolimits_{{i = 1}}^{n} I\left( {\vec{r}_{i} } \right) \cdot \vec{r}_{i} }}{{\mathop \sum \nolimits_{{i = 1}}^{n} I\left( {\vec{r}_{i} } \right)}}$$

with $\overrightarrow{r}$ the spatial position, $I$ the intensity value between 0 and 1 and $n$ the number of voxels in the 3D image. The calculated CoMs are visualized in Fig. 3B. The actual position of the micro-robot may differ from the position calibrated by the system matrix due to e.g. misplacement of the object in the sample holder. Therefore, the center position of the micro-robot was calibrated for each spatial direction independently by using the reconstructed images corresponding to a spatial position of the micro-robot in the zero plane of x-, y- and z-direction. These calibrated actual positions have been averaged and were then subtracted from all the calculated CoMs as a global offset. The accuracy was calculated as the mean euclidean distance between the evaluated CoMs and the calibrated actual positions of the micro-robot

$$\mathrm{a}\mathrm{c}\mathrm{c}\mathrm{u}\mathrm{r}\mathrm{a}\mathrm{c}\mathrm{y}=\frac{\sum _{i=1}^{N}||{\overrightarrow{r}}_{\mathrm{C}\mathrm{o}\mathrm{M}}^{i}-{{\overrightarrow{r}}_{\mathrm{a}\mathrm{c}\mathrm{t}\mathrm{u}\mathrm{a}\mathrm{l}}^{i}||}_{2}}{N}$$

with $N=27$ the number of frames.

### Navigation of the micro-robot with an MPI scanner

Assuming a direction of movement $\overrightarrow{v}$, defined by the azimuth angle $\mathrm{\phi }$ and the polar angle $\mathrm{\theta }$ in spherical coordinates, the direction of movement reads:

$$\vec{v} = \left( {\begin{array}{*{20}c} {{{\cos}} \, \phi \, {{\sin \, \theta }}} \\ {\sin \, \phi \, {{\sin \, \theta }}} \\ {{{\cos \, \theta }}} \\ \end{array} } \right).$$

The two vectors, perpendicular to $\overrightarrow{v}$, define the plane of rotation for the magnetic field vector:

$$\vec{B}_{1} = B_{{{\text{rot}}}} \left( {\begin{array}{*{20}c} { - {{\sin}} \, \phi } \\ {\cos \, \phi } \\ 0 \\ \end{array} } \right),\vec{B}_{2} = B_{{{\text{rot}}}} \left( {\begin{array}{*{20}c} {\cos \, \phi {{\cos \, \theta }}} \\ {\sin \, \phi \, \cos \, {{\theta }}} \\ { - {{\sin \, \theta }}} \\ \end{array} } \right)$$

with ${B}_{\mathrm{r}\mathrm{o}\mathrm{t}}$ the amplitude. A rotating field vector with an angular frequency $\mathrm{\omega }$ has the form:

$$\vec{B}_{{{\text{rot}}}} \left( t \right) = \vec{B}_{1} {{\cos~}} \, \omega t + \vec{B}_{2} ~\sin \, {{\omega }}t = B_{{{\text{rot}}}} \left( {\begin{array}{*{20}c} { - {{\sin}} \, \phi {{\cos~}} \, \omega t + \cos \, \phi \, \cos {{\theta }} \, {{\sin~}} \, \omega t} \\ {\cos \, \phi \, {{\cos~}} \, \omega t + {{\sin~}} \, \phi \, \cos {{\theta \, \sin }} \, \omega t} \\ { - \sin \, {{\theta \, \sin }} \, \omega t} \\ \end{array} } \right) = \left( {\begin{array}{*{20}c} {B_{x} } \\ {B_{y} } \\ {B_{z} } \\ \end{array} } \right).$$

The currents, which need to be applied to the three focus field coils of the MPI scanner are proportional to ${B}_{x}$, ${B}_{y}$ and ${B}_{z}$, respectively. The used MPI scanner (Bruker BioSpin) allows a maximum focus field strength of 18 mT. The application of slow alternating currents to the focus field coils is possible. For a movement of the micro-robot only in the horizontal plane (${\theta }={90}^{\circ }$) the rotating magnetic field simplifies to

$$\vec{B}_{{{\text{rot}}}} \left( t \right) = B_{{{\text{rot}}}} \left( {\begin{array}{*{20}c} { - {{\sin}} \, \phi \, {{\cos }}\omega t} \\ {\cos \, \phi \, {{\cos~}} \, \omega t} \\ { - {{\sin }} \, \omega t} \\ \end{array} } \right).$$

**Figure 8:**
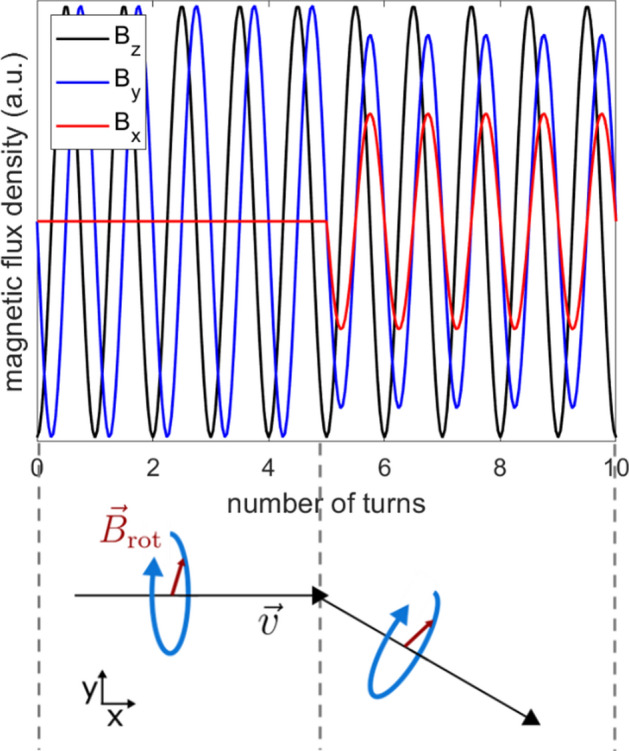
Magnetic actuation with an MPI scanner by using rotating focus fields. The resulting field vector ${\overrightarrow{B}}_{\mathrm{r}\mathrm{o}\mathrm{t}}(t)$ needs to rotate in the plane perpendicular to the direction of movement $\overrightarrow{v}$ of the micro-robot. Such a field vector is generated by the superposition of orthogonally generated focus fields of an MPI scanner.

A schematic drawing, showing the rotating magnetic field vector dependent on the direction of movement as well as the corresponding magnetic fields ${B}_{x}$, ${B}_{y}$ and ${B}_{z}$ can be found in Fig. 8.

The path segments are chosen from the CAD model of the phantom by placing five points along the middle cerebral artery. From the five points the intended direction of movement is calculated for each path segment giving ${\overrightarrow{B}}_{\mathrm{r}\mathrm{o}\mathrm{t}}(t)$ for four path segments. The number of turns have to be chosen according to the velocity of the micro-robot, the applied frequency and the path-length. The aneurysm phantom was filled with a red stained water-glycerol mixture with a mixing ratio of 2:1. The fluid had a viscosity of about 3.5 mPa·s (according to Cheng et al.^52^) to better imitate the conditions of blood. The micro-robot was inserted into the phantom and the openings were sealed. At a frequency of 5 Hz 38 turns in total resulting into an actuation time of 7.6 s were found to be suitable to cope the pathway with the micro-robot. Each period was discretized into 20 focus field steps, which can be read in by the MPI scanner. A ramping at the beginning and at the end within 10 focus field steps have been added to avoid large amplitude changes. That gives a total of 780 focus field steps. The ramping time between each focus field step was set to 10 ms, resulting into a rotation frequency of 5 Hz.

### Tomographic visualization and navigation with an MPI scanner

The angiogram and sequential images of the micro-robot have been acquired using the same preclinical MPI scanner as above (see “Tracking accuracy of the micro-robot”). The drive field amplitudes, gradient strength and system matrix shape have been constant throughout this work.

The aneurysm phantom was filled with tracer material (Resovist, 0.5 mmol Fe/ml, I’rom Pharmaceuticals, Tokyo, Japan) with a dilution of 1:100 for acquiring the angiogram. The angiogram was acquired within 15 s, such that the receive signals have been averaged 700 times. For image reconstruction a system matrix with a sample volume of 4 µl and a dilution of 1:3 of the same tracer material was recorded. Image reconstruction has been performed using frequency components of the system matrix ≥ 65 kHz that feature SNR values above 10. A Kaczmarz algorithm with 10 iterations and a Tikhonov-regularization with a regularization factor of 10^–3^ have been used. An intensity value threshold of 10% has been applied prior to visualization.

For visualizing the micro-robot within the aneurysm phantom during MA, the aneurysm phantom has been carefully rinsed and filled with the same water-glycerol mixture as for the navigation experiments. Then, the micro-robot has been inserted in the aneurysm phantom (see Fig. 4, first position). Since the micro-robot’s movement is approximated as a movement in the plane, the aneurysm phantom had to be rotated a little in comparison to the angiogram measurement. During MA, an MPI measurement was performed at the beginning and after each manipulation step. The measurements have been reconstructed using the system matrix described above (see “Tracking accuracy of the micro-robot”). Frequency components featuring minimum SNR values of 10 have been selected for reconstruction. A Kaczmarz algorithm with one iteration and a Tikhonov regularization with a regularization factor of 0.1 have been used. For visualization and CoM calculation, an intensity value threshold of 50% has been applied.

As stated above, the aneurysm phantom had to be rotated before MA. Therefore, the reconstructed image of the aneurysm phantom is rotated against the reconstructed images of the micro-robot within the aneurysm phantom. A registration is carried out by rotating the reconstructed 3D volume of the aneurysm phantom before MA. The superposition of the (rotated) angiogram and the reconstructed images of the micro-robot are visualized in Fig. 5 (top). Furthermore, the CoM of the reconstructed images of the micro-robot are superposed to the angiogram in Fig. 5 (bottom).

### Hyperthermia of micro-robot

The micro-robot is placed inside an Eppendorf tube with 16 µl water, such that the micro-robot is completely covered with water. Hyperthermia measurements are conducted with a self-built setup featuring a magnetic field of 557.5 kHz with 30 mT amplitude and a water shield absorbing the heat of the excitation coil. Additional polystyrene insulation separates the sample chamber from the water shield. A fiber optical temperature sensor measured the temperature of the water surrounding the micro-robot and two additional sensors measure the temperature of the excitation coil and the interface between the water shield and the insulation.

### Ethical approval and informed consent

All methods were carried out in accordance with relevant guidelines and regulations. The use of the clinical data was approved by the institutional review board of the University of Lübeck (registry number 20-121a) in this single-center retrospective study with waived individual consent. Informed consent was waived by the same ethics committee that approved the study.

## Supplementary Information

Supplementary Legend.

Supplementary Movie 1.

Supplementary Movie 2.
