# Supplementary material for: Navigation of a magnetic micro-robot through a cerebral aneurysm phantom with magnetic particle imaging
Source: Sci Rep. 2021 Jul 7;11:14082. doi: 10.1038/s41598-021-93323-4 (PMC8263782; doi:10.1038/s41598-021-93323-4)
Supplement: Supplementary file 1 — Supplementary Legend. [file 41598_2021_93323_MOESM1_ESM.docx]

Navigation of a magnetic micro-robot through a cerebral aneurysm phantom with magnetic particle imaging

Anna C. Bakenecker, Anselm von Gladiss, Hannes Schwenke, André Behrends, Thomas Friedrich, Kerstin Lüdtke-Buzug, Alexander Neumann, Joerg Barkhausen, Franz Wegner, Thorsten M. Buzug

Supplementary Information

**Numerical simulations on the micro-robot’s velocity**

Numerical simulations on the micro-robot’s velocity were performed with Comsol Multiphysics (Version 5.4, COMSOL AB, Stockholm, Sweden) by using two interfaces: Laminar Flow and Solid Mechanics. Laminar Flow was used to simulate the boundary conditions of the environment. Solid mechanics describes and calculates the velocity of the object in the medium, here water was used as the surrounding medium. A rotation of the micro-robot around its long-axis was applied. A step size of 0.01 s was used. The velocity of the translational motion of the micro-robot due to its rotation was measured. It was necessary to ramp up the rotational speed within 0.3 s toward 5 Hz. Fig. S1 shows a constant velocity of (1.56±0.11) mm/s in the regime of constant rotation frequency. It needs to be mentioned that the swimmer was simulated without the surrounding of a phantom or vessel wall, which is assumed to cause friction.


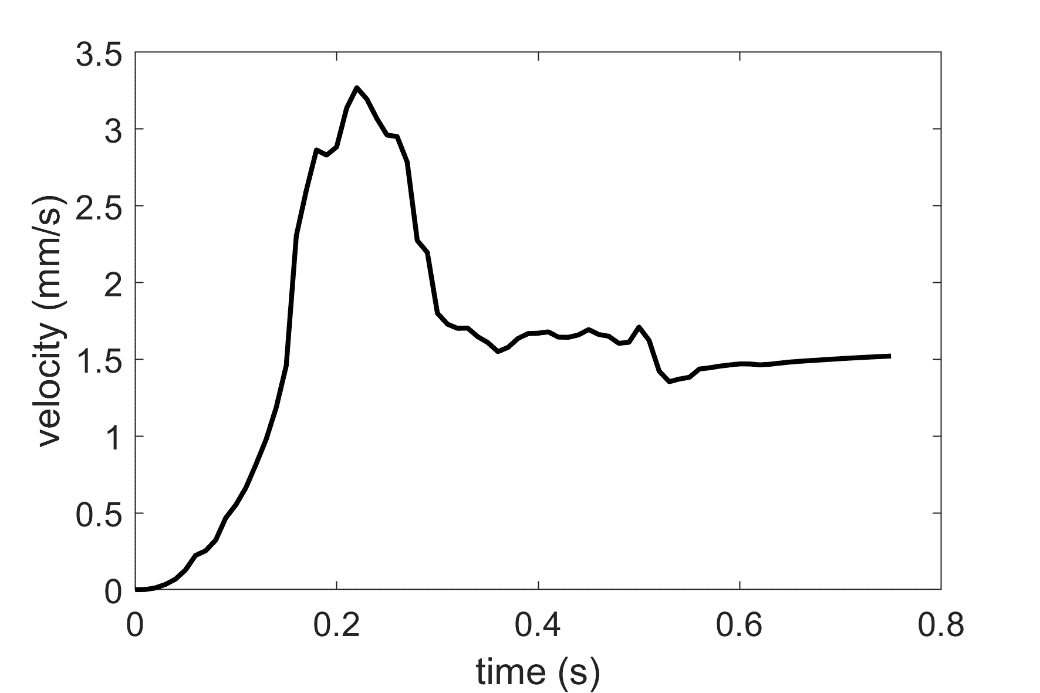


Fig. S1. Numerical simulations on the micro-robot’s velocity

After increasing the rotation frequency within 0.3 s, the micro-robot shows an approximately constant velocity of (1.56±0.11) mm/s at a rotation frequency of 5 Hz for a movement in water.

**Experimental investigation of the micro-robot’s fluid dynamics**

The velocity of the micro-robot was investigated by using a tabletop setup, which consist of two pairs of Helmholtz-Coils generating a rotating magnetic field vector. The magnetic micro-robot was placed inside a 3D-printed (Form3, Formlabs Inc.) channel of 24 mm length and 2 mm diameter. The channel was filled with the same glycerol-water mixture as the aneurysm phantom. Amplitudes of 3, 5 and 7 mT and frequencies between 2.5 Hz and 22.5, 35 and 40 Hz were applied, respectively. The velocity was measured four times (two times travelling in each direction) for each pair of frequency and amplitude. The movement was video recorded and the traveling time was evaluated by the videos. The results can be found in Fig. S2. A linear relationship between the applied frequency and the resulting traveling speed was expected up to a certain frequency, at which the viscous drag force becomes larger than the magnetic torque. This can be approximately observed for frequency regimes of up to 15, 25 and 30 Hz, respectively, where maximum velocities of 2.6, 3.8 and 4.5 mm/s were found. Afterwards the velocity drops rapidly, and a slip-stick movement was observed. Since the magnetic torque scales with the applied magnetic field strength, larger synchronous motion regimes were expected for larger field strength. This can be clearly observed.


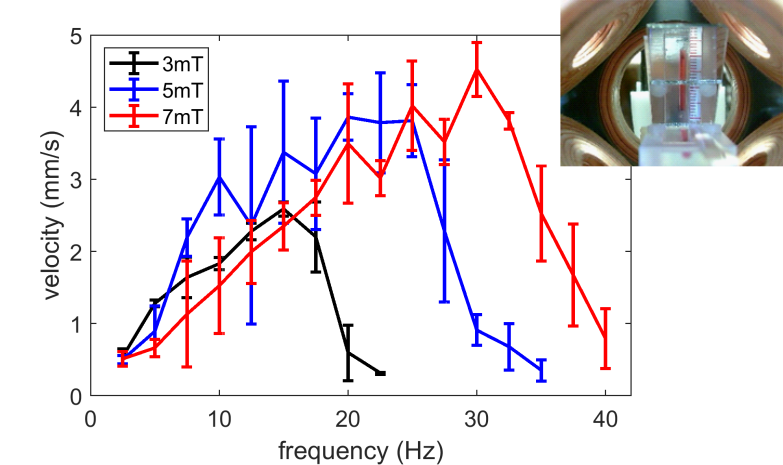


Fig. S2. Experimental measurement on the micro-robot’s velocity

The velocity was measured in a water-glycerol mixture (3.5 mPa·s), the same which was used inside the aneurysm phantom. A rotating field with a flux density of about 3, 5 and 7 mT was applied with two pairs of Helmholtz coils. A transition between the synchronous movement regime and the slip-stick movement was found to be at 15, 25 and 30 Hz, respectively, where maximum velocities of 2.6, 3.8 and 4.5 mm/s were found. The indicated error bars are expressing the standard deviation.

Movie S1.

Magnetic actuation of the micro-robot through the aneurysm phantom inside an MPI scanner.

Movie S2.

Sequential imaging and actuation of the micro-robot with an MPI scanner.
